# A Retrospective Study on Bovine Tuberculosis in Cattle on Fiji: Study Findings and Stakeholder Responses

**DOI:** 10.3389/fvets.2018.00270

**Published:** 2018-10-26

**Authors:** Elva Borja, Leo F. Borja, Ronil Prasad, Tomasi Tunabuna, Jenny-Ann L. M. L. Toribio

**Affiliations:** ^1^The University of Sydney, Farm Animal Health, Sydney School of Veterinary Science, Sydney, NSW, Australia; ^2^Vet Essentials, Suva, Fiji; ^3^Ministry of Agriculture, Koronivia Research Station, Koronivia, Fiji

**Keywords:** bovine tuberculosis, disease control, surveillance, BTEC, Fiji

## Abstract

Bovine tuberculosis (bTB) is globally significant due to its impacts on cattle production. A Brucellosis and Tuberculosis Eradication and Control (BTEC) program commenced in Fiji during the 1980's and has since been sustained by government funding and industry cooperation. A retrospective study of bTB data obtained during the Fiji BTEC program from 1999 to 2014 was undertaken at the University of Sydney with support from the Government of Fiji. It confirmed that bTB is well-established in dairy cattle farms in Naitasiri and Tailevu provinces of Central Division on the main island of Viti Levu, and suggested that the disease is present among cattle on farms in all or most provinces across three (Central, Northern, Western) of the four divisions in the country. It was evident that despite sustained efforts, disease reduction and containment was not being achieved. Reasons contributing to this situation included the appropriateness of the protocol for conduct of the single intradermal test (SID) in cattle, absence of regular quality assurance training of BTEC field staff, lack of standard procedures for bTB data collation and evaluation, unregulated cattle movements and the presence of stray cattle. The Fiji Ministry of Agriculture responded proactively to these findings by implementing revision to the use of the SID in cattle and refresher training for staff along with the Biosecurity Authority of Fiji who implemented cattle movement restriction. A subsequent apparent outbreak of bTB in some farms due to increased detection by the new test protocol raised concerns for the local dairy industry. To clarify the status and extent of bTB infection and the challenges faced by the industry, a stakeholder forum was held in May 2017, and a new BTEC strategy was formulated and endorsed by stakeholders. bTB remains a focus for cattle disease control by the government of Fiji. This case study highlights the challenges for bTB control in Fiji and underlines the importance of technical and social considerations to achieve success in disease control.

## Introduction

Bovine tuberculosis (bTB) is a chronic bacterial disease of cattle caused mainly by *Mycobacterium bovis*, although other zoonotic members of the *M. tuberculosis* complex may be the cause, such as *M. caprae*, the common cause of bTB in central Europe (1). bTB results in serious economic losses for the livestock industry worldwide due to animal disposal, carcass confiscation, premature culling, low production and poor reproductive performance (2). Further infection in people results in disease that is predominantly extra-pulmonary but cannot be clinically distinguished from *M. tuberculosis* infection. Official estimates of human zoonotic TB cases due to *M. bovis* in 2016 stand at 147,000 new cases and over 12,500 deaths, mainly in Africa and South-East Asia (3).

In Fiji, bTB leads to decreased production and opportunity for local trade due to sick animals and early culling of potentially productive stock. The bTB situation in Fiji is becoming an increasing concern for industry stakeholders as the culling of stock further aggravates the low milk production in the country. Data in 2014 from cattle sent to slaughter after a positive skin test on farm showed that one in three reactors (animals with positive single intradermal test) had generalized TB, and 85% had some form of gross TB lesion at post-mortem examination. Successful eradication of bTB is recognized by the Government of Fiji to be of benefit to individual cattle owners and to the country in relation to trade and potentially to human health. Hardest hit is the dairy sector which has suffered the greatest loss of cattle numbers (4).

This paper provides a case study of bTB control in an endemically infected cattle population in the Pacific. It outlines the bTB control program in Fiji, presents the methods and results of a retrospective study of bTB from 1999 to 2014 in Fiji, discusses the actions of the Ministry of Agriculture and other government agencies in response to study findings, and considers the implications of this response for industry, and longer-term, for the eradication program. As bTB remains a high priority for cattle disease control by the Government of Fiji, this case study highlights the challenges for bTB control in Fiji and underlines the importance of technical and social considerations to achieve success in disease control.

## bTB control in fiji

It is likely that bTB was introduced in Fiji through cattle brought in by European settlers during the 1830's (5). During the 1970's the deleterious effect of brucellosis and tuberculosis in local cattle farms was recognized and the need to establish a national control program voiced (6). Subsequently the Ministry of Agriculture (MOA) commenced the Fiji Bovine Brucellosis and Tuberculosis eradication and control (BTEC) program in the early 1980's with support from the Australian Government (6, 7) implementing dairy farm registration, cattle movement monitoring, and mandatory bTB testing and ear tagging of tested cattle, and carcass inspection at abattoir with compensation paid for condemnations at slaughter. These activities were based on property identification, animal tagging and surveillance programs of Australia (8) and the United Kingdom (9). However, the requirement for annual cattle farm registration is limited to dairy farms as the basis for legal sale of milk and milk products, with only some beef herds being voluntarily registered. Field testing was conducted annually although inconsistently between farms. Historical documentation on the BTEC program and bTB occurrence in Fiji is sparse with no information available prior to 1999. For example, the Animal Health Survey published in 1999 by the Secretariat of the Pacific Community (10) did not include a report for Fiji. Information about Fiji bTB from 1999 is limited to government reports and record books, and data reported to the World Organization for Animal Health (OIE) since Fiji became a member in 2007.

Cattle farms of all types (dairy and beef farms of individual farmers, school farms, village/settlement, government stations, middlemen) are included in the program and participation is mandatory although some farmers do not comply. There are no specific consequences for non-compliance other than ongoing transmission among cattle in non-compliant infected farms. All cattle aged 6 months and above are tested and have a metal tag with a unique number placed in the right ear to indicate the animal has been tested. The single intradermal test (SID) using purified protein derivative antigen from *M. bovis* (PPD-B) is administered at the caudal fold of the tail (CFT) with the result read 3 days after administration. Up to September 2014 a positive result was determined by the presence of a wheal not <4 mm in diameter. All log books and handwritten data collected from the field were filed by the Fiji Ministry of Agriculture in a government stock room. There was no written protocol for standard data management and analysis, and no systematic analysis of data to evaluate progress of bTB control over time. Quarterly and annual reports were prepared based on manual counts of records. Designated responsibility for the conduct of the bTB program was at the level of the division offices from 1999 to 2010 in an effort to increase surveillance coverage. This was centralized to the national office from 2011 to improve monitoring of the quality of testing.

Abattoir monitoring consists of carcass inspection for tubercle lesions by government meat inspectors at the two main abattoirs of the Fiji Meat Industry Board (FMIB) located in Nasinu, Central Division and Vuda, Western Division, respectively (11). Affected organs or whole carcasses are condemned based on the severity and location of tubercle lesions. Compensation to farmers is paid at slaughter of affected animal at a rate of FJD$1.60 per kg for the condemned part of the carcass and applies to animals detected through on-farm testing (reactors sent to slaughter) and to animals detected via carcass inspection at slaughter. Thus, this compensation is available to both farmers that comply with on-farm testing and those that do not. During 2015 the compensation rate was improved to equal the market price at the time of culling (12).

Farms with positive animals determined by on-farm testing or abattoir monitoring are classified as “Infected.” BTEC requires an infected farm to be free from bTB for 3 consecutive SID tests held at a minimum of 3-months intervals to obtain “Restricted,” “Provisionally clear,” and “Clear” statuses, respectively. It requires a minimum of 9 months from the time of detection for a farm to complete three consecutive clear tests and obtain “Clear” (bTB-free) status.

The Fiji BTEC program, a long-term activity sustained by annual government funding and industry cooperation, demonstrates collective commitment to address bTB in the cattle population. To underpin a review of the BTEC program, a retrospective study of bTB surveillance data from 1999 to 2014 was conducted over 12 months during 2014–2015. The aim of the retrospective study was to document the progress of the BTEC program and to provide recommendations to strengthen it. The final results of the study were formally presented to Ministry of Agriculture in September 2016 and to the industry stakeholders during the BTEC Forum held in March 2017.

## Retrospective study

### Materials and methods

#### Data sources

This study was conducted using data collected by the BTEC program from 1999 to 2014. The Fiji Ministry of Agriculture granted approval for use of the Fiji BTEC data to conduct this study in March 2014. Hard copies of batch books, reactor books, field sheets, annual reports, memorandum and other documents related to bTB in Fiji were used to collate and cross-check data from 1999 to 2014. The dataset compiled by year included farm identification number, location, farm type, date of testing, total number of cattle tested, total number of cattle test positive and farm TB status. For 2011 to 2014, the dataset for each year listed tests conducted by individual animal tag number. The few bTB test results from species other than cattle (horse, pig) were excluded, as were farm record data on the number of cattle younger than 6 months. It was assumed that all TB test results were read 3 days after the date of tuberculin administration.

Records of carcass inspection at slaughter from 2011 to 2014 were obtained for FMIB abattoirs at Nasinu and Vuda. Individual cattle records for slaughtered bTB positive animals were identified, including reactors identified during on-farm testing and subsequently sent for slaughter, and other animals identified at slaughter via detection of lesions during carcass inspection. The dataset compiled included farm identification number, animal identification number, date of slaughter and type of lesion detected. Complete records were only available for the Nasinu abattoir.

Due to the absence of a formal national registration system for all cattle farms, no absolute total cattle number were available for use as a denominator to calculate the population coverage of testing or infection prevalence in this study. In place of this, cattle population estimates for 2011 to 2014 published in the World Animal Health Information Database (WAHIS Interface) (13) were used. However, no reliable cattle population numbers at the national and division level were available prior to 2011.

#### Data transcription and sorting

For each year from 1999 to 2014, data were transcribed from hard copy sources into a purpose-built spreadsheet in Microsoft Excel version 2003. *Farm ID spreadsheet* included farm registration number, farm name, farm location (division, province, district, village/settlement), farm type (dairy, beef, other), date of test, number of cattle on farm by age group, number of animals tested, number of animals tested positive, number of animals tested negative and TB status of farm. *Animal ID spreadsheet* included farm registration number, farm name, farm location (division, province, district, village/settlement), date of test, TB tag number, age-gender description (heifer, dry cow, lactating cow, bull, steer), and TB test result.

Data transcription was performed by BTEC personnel from May 2014 to May 2015. Data sorting and validation conducted by the first author produced a comprehensive inventory of cattle farms and farmers from 1999 to 2014. This was verified for dairy farms by matching farm registration number and farm name to the MOA dairy farm registration list, and for beef farms based on familiarity of BTEC staff with farmers and farm operations as there is no formal registration system for beef farms. The list identified 2,141 cattle holding facilities (dairy and beef farms of individual farmers, school farms, village/settlement, government stations, middlemen) including subsistence or irregular cattle farm operations. When needed, missing values for farm location were entered based on recall. This list was sent to MOA Economic Planning and Statistics Division (EP&S) to validate location details recorded for each farm. To ensure that all testing data were for bTB SID tests, a cross-check against records for bovine brucellosis testing was performed.

#### Data analysis

*Farm ID* data from 1999 to 2014 and *Animal ID* data from 2011 to 2014 were available for analysis. Descriptive statistics for the number of positive farms and animals were calculated, and the number of positive animals detected through on-farm surveillance and abattoir monitoring were tabulated separately by division and by province per year.

*Farm ID* data were also analyzed to determine the number of tests conducted on infected farms each year and the status of each infected farm by year end. Within a calendar year, farms that had undergone one test with at least one reactor (SID test positive animal) were designated as “Infected.” Farms that had undergone one test round with at least one positive result and undergone one follow up test within the same calendar year with no positive result were designated as “Restricted.” Farms that had undergone two and three consecutive test rounds without any positive results were designated as “Provisionally clear” and “Cleared” farms, respectively.

For 2011 to 2014, the status of Infected farms from one calendar year to the next was investigated to identify farms that had positive cattle detected over consecutive years and did not attain cleared status within a period of two or more consecutive calendar years.

### Results

**Figure 1:**
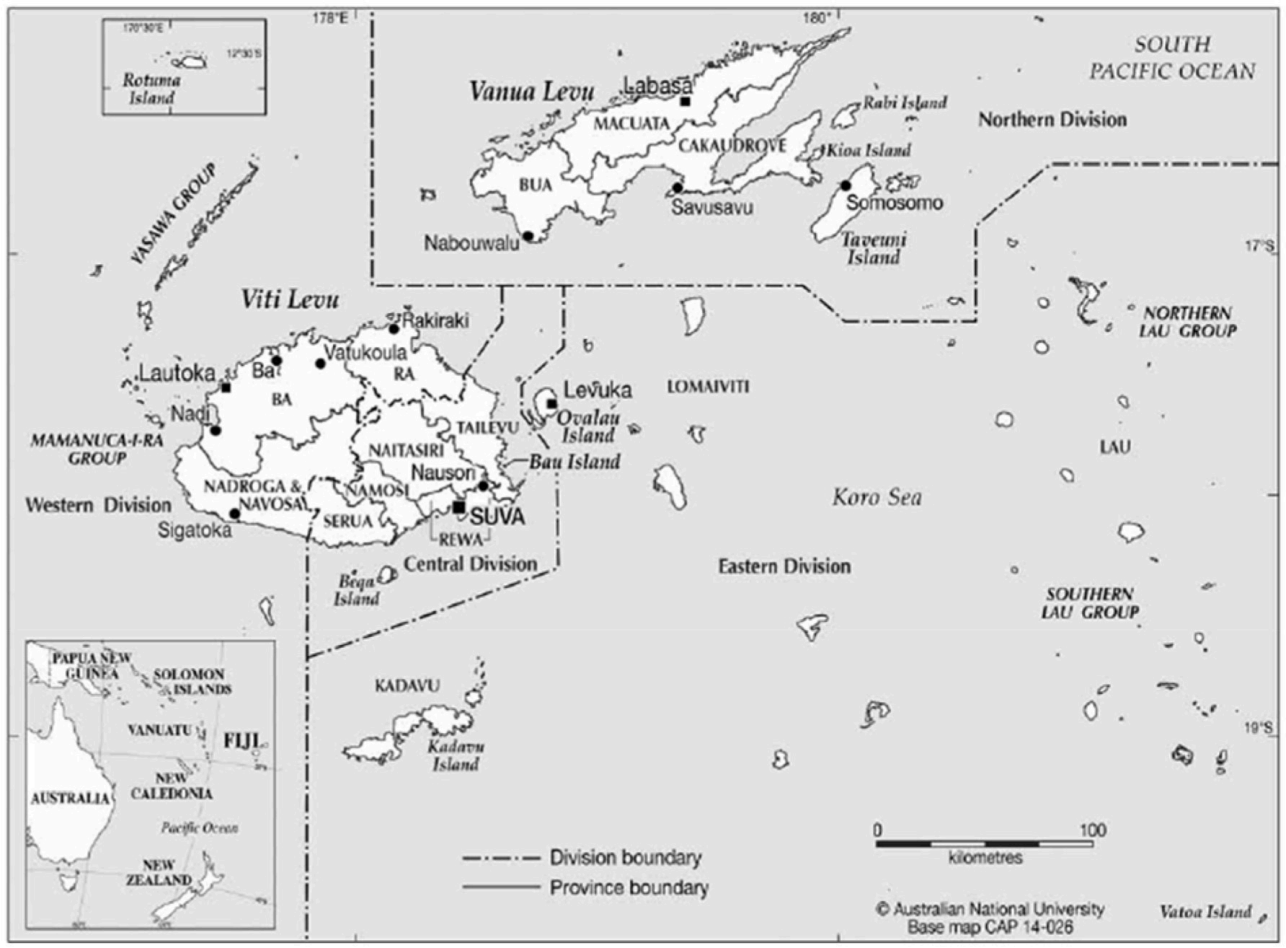
Map of Fiji showing Division boundaries. Source: Maps Online, CartoGIS Services, ANU College of Asia and the Pacific, The Australian National University.

From 1999 to 2014 ~2,141 cattle holding facilities were included in the BTEC program across the 4 divisions of Fiji (Figure 1). On average, 25,693 cattle (median: 27,562, range: 7,552–43,516) from 258 farms (median: 272, range: 96–438) were tested per year during these 16 years. The majority of animals tested were located in the Central Division with an average of 21,339 cattle (median: 25,102, range: 4,701–34,955) tested in this division every year from 1999. Less testing was undertaken elsewhere, with number of years testing conducted and total cattle numbers tested per division being for Western Division (16 years; median: 3,155, range: 139–9,064), Eastern Division (9 years; median: 28, range: 0–397) and Northern Division (6 years; median: 0 range: 0–2,265).

#### Coverage of fiji's BTEC program

Cattle population numbers reported to the FAO and OIE required to estimate population coverage by the BTEC program were deemed unreliable prior to 2011 because the process used to estimate the reported numbers was not documented. For 2011 to 2014, the cattle population reported to the OIE based on data collated by a government veterinarian ranged between 40,008 and 44,388 cattle and the percentage of cattle tested ranged between 33.6 and 74.0%, with variation between years arising mainly from changes in the number of cattle tested. The total tested was markedly lower in 2004, 2006, 2007, 2010, 2011, and 2013, and for 2010 this aligned with a lower budget allocation compared to the previous year (Table 1).

**Table 1:** Budget for the Fiji BTEC program^a^ and the number of farms and cattle tested for bovine tuberculosis by the program from 1999 to 2014.

**Year**	**BTEC budget (USD)**	**Number of**		**Number and percentage with positive result**			
		**Farms tested**	**Animals tested**	**Farms**		**Animals**	
				**No**.	**%**	**No**.	**%**
1999	36,450	373	38,870	56	15	220	1
2000	36,450	245	29,303	37	15	230	1
2001	72,900	299	26,277	50	17	293	1
2002	72,900	228	30,880	31	14	183	1
2003	72,900	170	27,506	26	15	121	0
2004	72,900	105	19,323	22	21	180	1
2005	72,900	438	41,591	34	8	192	0
2006	72,900	96	7,552	27	28	186	2
2007	114,079	98	9,569	23	23	61	1
2008	85,335	377	43,516	43	11	212	0
2009	718,065	417	32,160	11	3	39	0
2010	96,228	113	14,967	7	6	17	0
2011	437,400	136	14,916	14	10	60	0
2012	370,641	303	27,618	15	5	47	0
2013	364,500	324	17,439	11	3	61	0
2014	729,000	401	29,597	32	8	721	2
TOTAL	3,425,548	4,123	411,084	439		2,823	

a *Budget listed is the annual total for bovine brucellosis and bovine tuberculosis activities in the BTEC program*.

#### bTB positive animals and farms

A total of 2,823 TB positive cattle were identified from 1999 to 2014 with an average number of 176 (median 181.5) reactors per year (Table 1). The lowest number of positive cattle in a year was 17 from 7 positive farms in 2010, and the highest 721 reactors from 32 positive farms in 2014.

**Figure 2:**
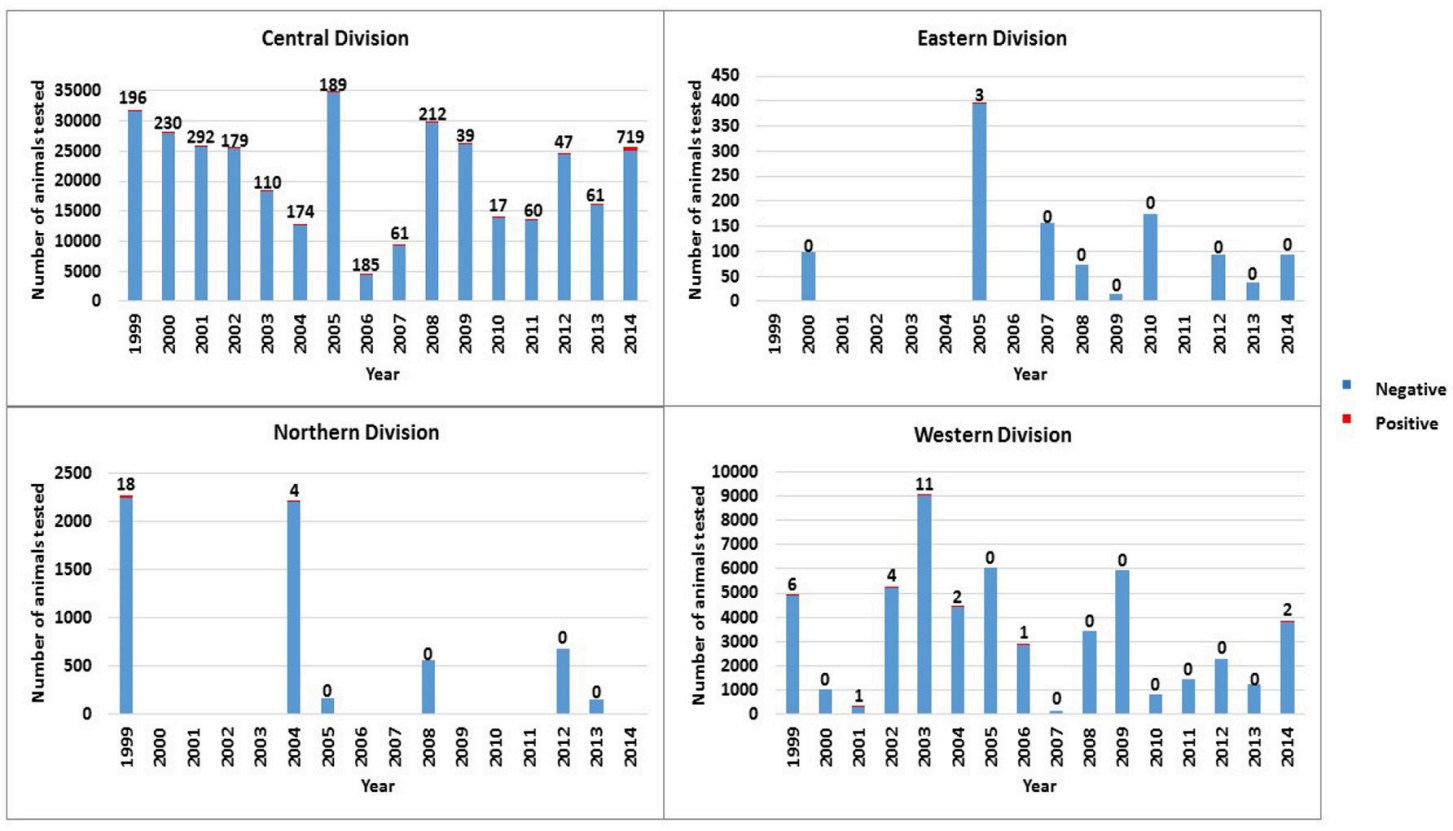
Total number of cattle tested and number of bTB test positive cattle by division per year from 1999 to 2014 in Fiji.

bTB positive cattle were identified in all four divisions of Fiji although the level of testing and the proportion of positive cattle varied between divisions (Figure 2).

For the Northern Division, testing was conducted in 1 of 4 provinces (Cakaudrove) in 6 years with positive cattle (termed reactors) detected in 1999 and 2004 (Figure 2). In 1999, 3 out of 9 (33%) farms tested in Cakaudrove province were positive (18 reactors). Testing was conducted only once on each positive farm and no further testing was scheduled in the Northern Division during the same year nor the following year to monitor the infected farms. In 2004, 1 out of 11 (9%) farms tested in the Northern Division was positive (4 reactors). This farm had been identified as infected in 1999 (15 of 18 reactors). No follow-up test was conducted to monitor this infected farm in 2004 or in 2005. No records were available to confirm if any of the reactors from the Northern Division were immediately culled.

In Eastern Division testing was conducted in 3 of 5 provinces (Kadavu, Lakeba Lau, Lomaiviti) in 9 years with three positive cattle from the two farms tested in Lomaiviti in 2005, and none in the other years (Figure 2). No further testing was conducted to monitor these two farms in 2005 or in 2006. No records were available to confirm if reactors were immediately culled from these farms.

In Western Division testing was conducted each year, and though usually undertaken in at least 2 of the 4 provinces annually for 16 years, most testing was conducted in Ba and Navosa/Nadroga provinces. Positive animals were detected in 7 of 16 years with the number and percentage of positive cattle ranging from 1 to 11 positive cattle or 0.04–2.27% (Figure 2). One farm was identified as positive in 2002 (all 4 out of total 4 reactors detected in this division in 2002 were located on this farm), 2003 (10 of total 11 reactors located on this farm), 2004 (2 of total 2 reactors located on this farm) and 2014 (2 of total 2 reactors located on this farm). No records were available from the abattoir at Vuda, in Western Division for this study to confirm if reactors were culled.

**Figure 3:**
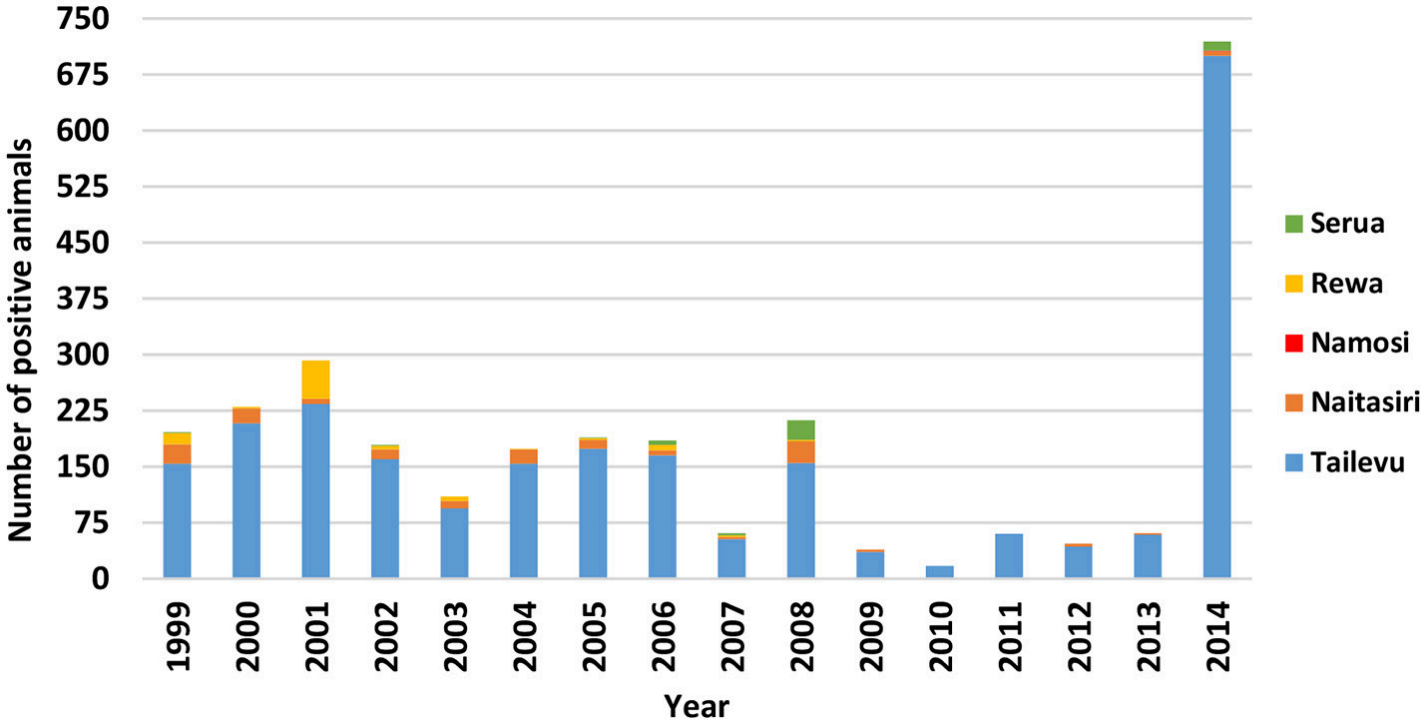
Number of bTB test positive cattle per year in the five provinces of Central Division in Fiji from 1999 to 2014.

For Central Division, testing was conducted each year in the 5 provinces (Naitasiri, Namosi, Serua, Tailevu, Rewa) with positive cattle detected consistently (Figure 2), particularly in Tailevu province that had test positive cattle each of the 16 years with the highest number of positives recorded in 2014 (Figure 3). Seven hundred reactors were detected in Tailevu from 23 of the 147 farms tested (15.7%) in the province during 2014.

For the 16 years that testing was conducted in Naitaisiri province, reactors were detected each year except in 2010 and 2011 when lower numbers of cattle were tested (862 in 2010, 1,833 in 2011). For the other years, higher numbers were tested with 7,046 animals (3 reactors) tested in 2009, 5,044 animals (4 reactors) tested in 2012, 4,466 animals (2 reactors) in 2013, and 7,831 animals (6 reactors) tested in 2014.

Serua province had the second highest number of reactors (12 of 721 reactors) in 2014, compared to earlier years when Naitasiri commonly ranked next to Tailevu. Data showed that in 2014, reactors in Serua came from only 1 of 6 farms (17%) tested in the province.

#### Farm types

An average of 168 dairy cattle (average: 168, median: 172.5, range: 16–717) were detected positive each year during the last 16 years compared to beef cattle (average: 3, median: 3, range: 0–18). Ninety-nine percent (2,685 of 2,690) of positive dairy animals detected from 1999 to 2014 were from Central Division.

For 1999 to 2014 in Central Division, a small proportion of dairy cattle tested positive every year (range 0.1–4.2%), from 0.1 to 1.5% of beef cattle tested positive in 8 of 16 years, and < 1% of cattle from other farm types tested positive in 10 of 16 years (Table 2). In Western Division, lower proportions of positive cattle were detected in 4 of 14 years that beef cattle were tested, in 3 of the 13 years that dairy cattle were tested, and in 2 of the 15 years that cattle from other farms were tested (Table 2).

**Table 2:** Number of cattle tested and of bTB test positive cattle by farm type from 1999 to 2014 in Central Division and Western Division Fiji.

**Year**	**Central Division**							**Western Division**						
	**% Of positive animals**			**Number of animals tested**				**% Of positive animals**			**Number of animals tested**			
	**Beef**	**Dairy**	**Other^a^**	**Beef**	**Dairy**	**Other^a^**	**Total**	**Beef**	**Dairy**	**Other^a^**	**Beef**	**Dairy**	**Other^a^**	**Total**
1999	0.2	0.7	0.3	919	23,598	7,195	31,712	0	0.5	0.1	982	396	3,515	4,893
2000	0	0.9	0.5	1,218	23,414	3,525	28,157	0	0	0	0	184	864	1048
2001	0.5	1.3	0.4	366	21,459	4,154	25,979	1.2	0	0	82	216	0	298
2002	0	0.8	0.3	662	23,064	1,935	25,661	0.1	0	0	4,900	0	319	5,219
2003	0	0.7	0.1	347	16,386	1,709	18,442	0.1	0.5	0	8,699	186	179	9,064
2004	0	1.4	0.9	387	12,110	214	12,711	0.2	0	0	1,029	17	3,363	4,409
2005	0.1	0.6	0	1,583	29,990	3,382	34,955	0	0	0	3517	132	2427	6076
2006	1.5	4.2	0	197	4,341	163	4,701	0	0.5	0	2,609	183	59	2,851
2007	0	0.7	0.4	304	7,662	1,308	9,274	0	0	0	0	0	73	73
2008	0.2	0.8	0	1,261	27,038	1,561	29,860	0	0	0	1,006	323	2,130	3,459
2009	0	0.2	0	904	24,559	716	26,179	0	0	0	1,695	4,134	97	5,926
2010	0	0.1	0.2	106	13,356	531	13,993	0	0	0	483	41	275	799
2011	0	0.5	0	453	12,776	263	13,492	0	0	0	1,102	0	322	1,424
2012	0.1	0.2	0.8	1,171	22,987	384	24,542	0	0	0	941	1,030	334	2,305
2013	0.5	0.4	0	560	15,173	286	16,019	0	0	0	122	1,037	68	1,227
2014	0.1	3.1	0.1	1,509	22,856	1,356	25,721	0.1	0	0	2,301	72	1,410	3,783

a *Farms with beef and/or dairy cattle that included school farms, villages/settlements, government stations and middlemen*.

Among the dairy cattle that tested positive from 2011 to 2014 in Central Division, a high proportion were productive female cattle, for example, in 2011 when all test positive animals were dairy cattle, 61.7% were dairy cows and a further 16.7% were heifers selected to be milkers (Table 3).

**Table 3:** Number of bTB test positive cattle by age-gender group from 2011 to 2014 in Central Division Fiji.

**Year**	**Heifer^a^**	**Dry cow^b^**	**Lactating cow^c^**	**Bull^d^**	**Steer^e^**	**No data**	**Total**
2011	10	26	11	9	4	0	60
2012	14	8	17	4	4	0	47
2013	16	12	27	5	1	0	61
2014	134	212	260	76	33	4	719
Total	175	259	315	94	42	4	889

a *Heifer, female at least 6 months of age and not yet mated*.

b *Dry cow, adult female more than 12 months of age not being milked at time of test*.

c *Lactating cow, adult female more than 12 months of age being milked at time of test*.

d *Bull, adult uncastrated male*.

e *Steer, castrated male at least 6 months of age*.

#### Classification of bTB infected farms

Data show that from 1999 to 2014, no farms were cleared of bTB infection within a calendar year (Table 4).

**Table 4:** Number of farms tested per division and total number of bTB positive farms from 1999 to 2014 with classification of these farms by the end of the calendar year.

**Year**	**Farms tested**	**Central**	**Eastern**	**Northern**	**Western**	**Total Positive**	**Infected^a^**	**Restricted^b^**	**Provisionally clear^c^**	**Clear^d^**
1999	373	49	0	3	4	56	47	9	0	0
2000	245	37	0	0	0	37	24	9	4	0
2001	299	49	0	0	1	50	40	8	2	0
2002	228	30	0	0	1	31	22	8	1	0
2003	170	24	0	0	2	26	24	2	0	0
2004	105	20	0	1	1	22	20	2	0	0
2005	438	32	2	0	0	34	20	9	5	0
2006	96	26	0	0	1	27	26	1	0	0
2007	98	23	0	0	0	23	23	0	0	0
2008	377	43	0	0	0	43	16	23	4	0
2009	417	11	0	0	0	11	8	2	1	0
2010	113	7	0	0	0	7	2	5	0	0
2011	136	14	0	0	0	14	12	2	0	0
2012	303	15	0	0	0	15	11	3	1	0
2013	324	11	0	0	0	11	10	1	0	0
2014	401	31	0	0	1	32	25	4	3	0
Total	4,123	460	2	4	11	439	330	88	21	0

a *Infected, farm with bTB positive cattle determined by on-farm testing or abattoir monitoring*.

b *Restricted, an infected farm after one negative round of testing*.

c *Provisionally free, an infected farm after two negative rounds of testing a minimum of 3 months apart*.

d *Clear, an infected farm declared bTB-free after three consecutive negative rounds of testing each a minimum of 3 months apart*.

Nine farms with positive cattle detected from 2011 to 2014 through on-farm testing and abattoir monitoring were all dairy farms situated in the localities of Waimaro and Namalata in Tailevu province (Table 5). Farms A and B had reactors consistently from 1999 to 2014. Except for Farms C and G, all other farms listed had their highest count of reactors in 2014. These farms are all located along an estimated 9.6 km stretch of the single major road in Tailevu.

**Table 5:** Number of bTB positive cattle per year for the nine dairy farms in Tailevu province that were consistently positive for bovine tuberculosis from 2011 to 2014 detected through on-farm testing and carcass inspection at the abattoir.

**Farm**	**Number of positive animals per year**															
	**1999**	**2000**	**2001**	**2002**	**2003**	**2004**	**2005**	**2006**	**2007**	**2008**	**2009**	**2010**	**2011**	**2012**	**2013**	**2014**
A	10	43	34	31	27	48	18	21	7	19	10	5	21	6	36	76
B	2	53	11	27	8	4	29	16	2	28	5	2	7	14	20	199
C	9	9	10	15	5	10	1	12	1	1	0	0	8	3	23	11
D	0	2	4	4	0	5	4	6	2	3	6	0	6	6	5	43
E	11	9	20	5	2	41	31	20	4	39	0	2	17	23	17	53
F	0	6	11	1	0	0	10	7	2	10	0	1	18	4	16	66
G	6	6	10	6	0	8	3	13	0	0	0	0	7	6	1	4
H	23	18	77	6	26	8	7	22	6	15	9	0	31	11	12	76
I	0	0	0	0	0	1	4	0	0	4	0	0	4	3	1	24

#### Case detection at carcass inspection

Each year from 2011 to 2014, cattle from Central, Western and Northern Divisions slaughtered at the FMIB Nasinu abattoir were found to have tubercle lesions during meat inspection (Table 6). The highest number of positive animals were from Tailevu province in Central Division with an average of 67 animals (268 total positives) detected in the abattoir per year. Although there was no reactor detected in Naitasiri during field testing in 2011 (Figure 3), three positive animals were detected at this abattoir. Further positive animals from the Northern Division were detected consistently from 2011 to 2014 with no on-farm detections despite testing conducted in 2012 and 2013 (Figure 2). Trace back of positive cattle from the Northern Division using the individual TB tag numbers showed that the 10 positive cattle had either read negative during on-farm testing or had never been tested on farm. This may imply that there are positive animals that are non-reactive to SID PPD-B affecting BTEC's proficiency in detection of infected animals in the field.

**Table 6:** Provinces with bTB positive cattle detected by meat inspectors at the FMIB Nasinu abattoir in Central Division, Fiji from 2011 to 2014.

**Division**	**Province**	**Number of positive animals detected in the abattoir**				
		**2011**	**2012**	**2013**	**2014**	**Total**
Central	Naitasiri	3	3	2	5	13
	Rewa	2	0	1	1	4
	Serua	1	0	0	0	1
	Tailevu	80	42	84	62	268
Northern	Bua	2	0	1	2	5
	Macuata	0	1	2	2	5
Western	Ba	1	0	1	0	2
	Navosa/Nadroga	0	6	6	2	14
	Ra	1	1	1	0	3
Grand Total		90	53	98	74	315

### Situation analysis and recommendations

The findings of the retrospective study confirmed that bTB had been endemic in Fiji for more than 16 years. Between 3 and 28% of farms tested per year in the BTEC program included cattle that tested positive to the SID test determined by the presence of a wheal size ≥4 mm until September 2014. This designation for a positive result at the highly specific interpretation of wheal ≥4 mm at the caudal fold was not adequate to identify sufficient positive animals for culling on infected farms to prevent ongoing bTB transmission.

There is clear evidence that bTB is well-established in the dairy cattle farms in Naitasiri and Tailevu provinces of Central Division on the main island of Viti Levu. While the strength of evidence for these provinces arises from a concentration of the BTEC program on-farm testing on the dairy farms in these two provinces, the abattoir monitoring results also support the conclusion of higher infection in these provinces at least for 2011–2014. Identification of SID test positive cattle in Central Division over multiple years also in beef farms (8 of 16 years) and other farm types (10 or 16 years) suggests that bTB infection is established throughout the cattle population.

Further the on-farm testing results and abattoir detections provide evidence that bTB is present among cattle farms in the other three divisions of the country, and in all 4 provinces of Western Division and in 3 of 4 provinces of Northern Division. Given the substantially lower numbers of dairy cattle in these other 3 divisions, this suggests that bTB is established at least among some beef cattle farms in Western Division and Northern Division.

From 1999 to 2014, the consistent positive status of a small number of farms and the fact that no farms were cleared of bTB infection within a calendar year (whilst acknowledging that a minimum of 9 months is required to progress from infected to clear status) is clear evidence that the test and cull plus quarantine procedures as applied for infected farms were inadequate to clear infection from a farm. The example of nine dairy farms located along one road in Tailevu province that were consistently positive for 2011–2014 exemplifies the situation with persisting infection.

The descriptive analysis of the BTEC data from 1999 to 2014 provided disturbing evidence that despite sustained efforts in on-farm testing and carcass inspection at abattoirs, BTB disease reduction and containment was not being achieved. This situation is well-illustrated although limitations of the 1999–2014 BTEC data, such as considerable variation in number of farms and animals tested between years and the positive SID designation based on wheal ≥4 mm, restricted the retrospective study to descriptive analyses.

Factors contributing to this situation and recommendations to strengthen the BTEC program are presented in Table 7. Further, given the need to identify bTB-free areas in Fiji that may be sources of replacement stock, surveillance sampling of farms in the provinces of Kadavu and Lakeba Lau in Eastern Division should be conducted to confirm if these areas are bTB-free and permit declaration of a bTB-free zone in the country (14).

**Table 7:** Factors contributing to this situation and recommendations to strengthen the BTEC program in Fiji.

**Factor**	**Related to**	**Main recommendations**
Insufficient consistency in the number and location of farms tested between years	Changes between years in government budget for the BTEC program eg reduction in 2006–2007 following political crisis in 2005/2006. Changes between years in budget allocation for bTB in the BTEC program eg reduction in 2009–2010 due to response to brucellosis detection after 13-years absence of detections (7). Insufficient number of BTEC field staff to conduct SID testing. No interrogation of BTEC records to inform plans for on-farm testing.	Ensure a consistent, adequate annual budget allocation for the BTEC program and the bTB component of it. Ensure adequate number of BTEC field staff. Implement a planning process for the BTEC program based on regular interrogation of bTB records with veterinary oversight. Establish a national database for data storage, manipulation and reporting.
Standard operating procedure for reading of SID test	Negative designation for any reaction at injection site <4 mm across all farms irrespective of status (unknown, infected, clear) will have led to a false negative result for some infected animals, such as cattle with chronic infection subsequently identified with tubercule lesions at abattoir carcass inspection and have impeded clearance of infection from infected farms.	Review of the SOP for reading of SID test particularly for known infected farms.
Inconsistent application of SOP for SID testing	Inadequate training and supervision of BTEC field staff.	Provide adequate training for BTEC field staff. Ensure adequate veterinarians in the BTEC program to supervise field staff.
Inconsistent application of SOP for test and cull and quarantine on infected farms	Inadequate training and supervision of BTEC field staff.	Provide adequate training for BTEC field staff. Ensure adequate veterinarians in the BTEC program to supervise field staff.
Unregulated cattle movements	Inadequate specification and implementation of cattle movement regulations.	Review of regulations on cattle movement administered by Biosecurity Authority of Fiji. Improve implementation of regulations by Biosecurity Authority of Fiji and consider involvement of harmonization with Ministry of Agriculture in implementation.
Stray cattle	Presence of stray cattle (untethered owned and unowned cattle grazing freely on public land and intruding on private land) acting to maintain infection in known infected areas.	Review of regulations on stray cattle administered by Biosecurity Authority of Fiji. Improve implementation of regulations by Biosecurity Authority of Fiji.

## Responses to findings of the retrospective study

The Fiji Ministry of Agriculture responded proactively to the findings of the retrospective study along with the Biosecurity Authority of Fiji (BAF) and the Fiji Cooperative Dairy Company Limited (FCDCL). The earliest responses commenced in late 2014 initiated following a preliminary analysis of the bTB records for 2011–2013. The response actions taken from 2014 to 2018 are described in detail below.

### SOP for on-farm testing

The MOA updated the 2010 BTEC SOP in September 2014 and consequently implemented re-training of staff and calibration of BTEC field testing equipment. The revised protocol identified reactors as all animals that developed any size of wheal or redness in the SID injection site at the caudal fold 3 days after administration of PPD-B, following the OIE recommendation for detection of reactors in known infected farms (1). On the assumption that all cattle in Fiji are potentially bTB infected, this new protocol was applied to all farms, regardless of whether farms were previously identified as disease-free or infected (15, 16). This SOP change was implemented to improve the sensitivity of detection of infected animals in the field. A change that was needed for example due to identification at carcass inspection of some cattle with tubercle lesions that had previously tested negative using SID. A subsequent apparent outbreak of bTB in some farms was due to increased detection by the new test protocol, with a total of 721 reactors from 32 farms in 2014 compared to 61 reactors from 11 farms in 2013 (Table 1). This event raised concerns for the local dairy industry. The extent of infection in these farms was confirmed by post-mortem inspection of bTB reactors. For reactors at slaughter at FMIB Nasinu abattoir in Central Division, the percentages with generalized TB, gross TB lesions and no visible lesions were 33, 51, 16% in late 2014 (*n* = 301), and 26, 40, 34% in 2015 (*n* = 1101), respectively (17). The dairy sector experienced the greatest loss of cattle due to culling of reactors, and this had a serious economic impact for individual farmers and for the industry leading to a shortage of dairy cattle in the country, a reduction in the volume of milk produced, and an increase in the volume of imported processed milk (4). Dairy farmers with smaller, semi-commercial farms slowly converted to cash crops to supplement their dwindling income. In response to this serious situation, the MOA improved its compensation scheme in August 2015 to match current market prices per kg of condemned carcass. The purpose was to assist farmers recover quickly after losses from bTB (12).

### Regulation of cattle movement

On 03 March 2016, as part of the disaster response of the Biosecurity Authority of Fiji post-Cyclone Winston a movement restriction on live animals was implemented to discourage movement of livestock without prior approval from the BAF or the Fiji National Disaster Management Office (18).

Subsequently on 13 January 2017 under section 77 of the Biosecurity Act 2008, the whole of Fiji was declared a biosecurity emergency area for Bovine Tuberculosis (*Mycobacterium bovis*) (19). During November 2017, the BAF and the MOA documented a movement control policy (20) to provide guidance on the implementation of cattle movement control. Movement of all calves and cattle within Fiji is strictly prohibited without prior authorization from BAF. Movement of cattle or calves without authorization is an offense attracting a maximum penalty of FJD 40,000 for individuals and FJD 200,000 for businesses or imprisonment. On 03 February 2018, the declaration was extended for continued implementation for a further 6 months.

### National stakeholder forum

To clarify the status and extent of bTB infection and the challenges faced by the industry and to promote communication and collaboration in delivery of the BTEC program, a stakeholder forum was held in May 2017 with government MOA, Biosecurity Authority of Fiji (BAF) and Ministry of Health (MOH) representatives, Fiji industry stakeholders and relevant experts from Australia and New Zealand. Presentations highlighted the needs for a clear policy and strategy for bTB eradication and rehabilitation, action to address overlapping and unclear legislative and stakeholder responsibilities (particularly between MOA and BAF), immediate removal of infected cattle from farms, auditing and capacity building programmes, and a data recording system for monitoring, evaluation and learning (4). Stakeholders agreed that the BTEC Program requires further investment from the government to set up a stronger team structure with necessary equipment for disease surveillance and personnel with appropriate legal powers to effectively undertake its field operations. A draft BTEC strategy was developed during the forum and endorsed by stakeholders, and members for the BTEC planning committee designated to finalize the strategy document.

### Documentation of fiji brucellosis and tuberculosis eradication strategy

The Ministry of Agriculture further refined and finalized this strategy in early 2018. Input to this process included review and recommendations on meat hygiene, bTB control strategies and diagnostic test selection by a technical team under the Government of Chile funded project “Strengthening the institutions responsible for the inspection and certification of agricultural products, and the coordination of the national system of food safety in Fiji” (21).

The goal of the Fiji BTEC Strategy is total eradication of bovine tuberculosis and brucellosis by 2037. The documented strategy lays out the direction for future implementation of the Fiji BTEC Program to attain the long term goal of official recognition by OIE of Fiji as free from both bovine brucellosis and bovine tuberculosis and maintaining this disease-free status (4). The strategy document includes specification on testing policy and strategy, zoning, reactor disposal and compensation, governance and operational management including staffing. It states a new role, full-time project manager, recognizing its importance for effective implementation of the BTEC program and an appointment effective June 2018 is being supported by the Fiji Dairy Industry Development Initiative [funded by New Zealand Ministry of Foreign Affairs and Trade (MFAT)].

### Further initiatives

Along with improvements to field testing and cattle movement control, opportunities for simultaneously improving the laboratory diagnostic capacity for bTB early detection and confirmatory diagnosis have been sought. The BTEC veterinarians and managers have established laboratory network links with Australia, India, New Zealand and Thailand to support diagnostic capacity building in Fiji. Under discussion with the World Organization for Animal Health (OIE) is funding for a Laboratory Twinning Program between FVPL and Animal and Plant Quarantine (QIA) Korea for proficiency testing and laboratory management training.

Concerned about the potential contribution of zoonotic TB to the human TB burden in Fiji, the MOA in collaboration with the Fiji Ministry of Health and Medical Services and the University of Sydney funded by the Marie Bashir Institute will undertake geospatial analysis of human tuberculosis cases and bTB-infected cattle farms, pilot TB surveillance of households in identified high risk areas for bTB exposure, and send samples from human extra-pulmonary cases and cattle cases for species determination. This investigation arises from concern about levels of extra-pulmonary tuberculosis (EPTB). During 2016 among the 312 notified human TB cases, 29% were classified as extra-pulmonary tuberculosis, nearly double the 15% of extra-pulmonary tuberculosis cases among the global total of human TB notifications in 2016 (3, 22). The contribution of bTB to these EPTB cases in Fiji is unknown because the routine diagnostics used do not distinguish pathogen species. There is suspicion of involvement due to the practice of raw milk consumption in some households that own cattle.

## Discussion

When the preliminary analysis of 2011–2013 bTB records indicated wide spread endemic infection, the Fijian government acted swiftly in September 2014 to revise SOP for SID testing. This was followed up by actions from 2014 to 2018 that have enhanced identification of infected cattle farms and removal of infected cattle, strengthened implementation of restrictions on cattle movements, and led to the endorsement of a new Fiji Brucellosis and Tuberculosis Eradication Strategy. These are critical steps on the journey to reduce bTB in the national cattle herd, and then subsequently to progress to bTB eradication. This staged process of bTB reduction and containment followed by eradication can be guided by the lessons learnt by other countries on the road to bTB control and eradication, such as Australia, Ireland and New Zealand. The generic components, first of bTB control and containment while ensuring continuity of the industry, and second of bTB eradication and proof of freedom must be contextualized to the bTB situation in Fiji. A policy based on contemporary scientific evidence and international best practice in bTB control needs to be accompanied by specific research in Fiji, given its particular geoclimatic and cultural features. It is crucial for the Fijian government and the dairy and beef industries to be aware that the current policy will need to be modified over time and the commitment to implementation maintained when the BTEC program transitions to the final eradication stage. Industry concern about an increasing proportion of SID test positive cattle with no visible lesions at slaughter is expected with continuation of current SOPs. This provides an example of a situation where technical expertise is required to inform future decisions on test protocol, and where specific research would be beneficial to determine if false positive cases are present and to understand the basis and the extent of these. The international community also needs to consider its role in supporting the Fijian government and industry to attain bTB freedom for the benefit of animal and human health in the Pacific. As Fiji serves as a regional hub, providing live animal stocks and animal products to the neighboring island countries, addressing bTB in Fiji supports the long-term goals of sustainable livelihood and food security in the Pacific island region.

The case study of bTB control in Fiji offers lessons within a Pacific context about the importance of the following technical and social aspects to achieve success in animal disease control.

1. Objective, ongoing assessment of bTB distribution using agreed performance measures (such as bTB farm incidence, reactors per thousand tests, number and proportion of reactors removed) is internationally accepted as essential for critical assessment of progress toward control and eradication (8). This requires a national database for data storage, manipulation and reporting plus data sharing with other national systems for cattle movement and farm registration. It is timely that the NZ MFAT funded project Fiji Dairy Industry Development Initiative has extended its project coverage to include development of web-based database which will link the BTEC geospatial and farm registration information with the agriculture census information of the Economic Planning and Statistics Division of the Ministry of Agriculture. Funding to progress has been approved and the database is now at the planning stage.
2. Robust and accurate diagnostics able to minimize false farm or animal designation given bTB prevalence level at the relevant stage of the control and eradication process must be applied. Selection of the most appropriate diagnostic test/s given the stage of control program and the field conditions for animal testing requires expertise in test protocols. Understanding is needed of the costs of false designation to bTB maintenance and spread (in relation to false negative animals/farms) and to unnecessary loss of productive animals and prohibition to trade (in relation to false positive animals/farms). The Fiji MOA recognizes that early identification of infected farms and infected animals is critical. To date the SID PPD-B in the caudal fold is the single diagnostic applied in the Fiji BTEC program due principally to its low cost and practical suitability to on-farm conditions. While the combination of SID test in the caudal fold (assuming use of potent tuberculin) and carcass inspection at slaughter is reasonable for detection of infected farms, a more sensitive testing regime is needed to support eradication from known infected farms. Thus, the MOA is considering application of other diagnostics, such as the interferon-γ test as a confirmatory test for SID positive animals in known infected farms, and increased use of culture to confirm status of lesions identified at abattoir carcass inspection. A cost-benefit analysis on the use of single intradermal comparative tuberculin test (SICTT) and interferon-γ [with sensitivity when applied in parallel approaching 93% (23)] in place of SID for cattle on known infected farms to aid control and eradication whilst maintaining a milking herd to permit business continuity is recommended.
3. Quality control (QC), the managerial process to compare actual and desired performance of a service or product, will act to ensure an animal disease control program is meeting its objective at the best possible return for the funds invested (24, 25). When disease detection is based on diagnostic procedures with aspects that have subjective interpretation, such as the SID PPD-B and post-mortem inspection (26), quality control will contribute to improve accuracy and consistency in detection. The Irish bTB eradication program with QC applied inputs (personnel, training, SOP, equipment, tuberculin, reagents, computerized recording system), performance (post-mortem surveillance, field surveillance) and outputs (test results, program delivery), provides a model for consideration. For example, the National Handbook of the Irish program that states the national policy and SOP for veterinary management of herds under restriction due to bTB is revised every 3 years to ensure continued improvement and refinement of program activities (27). Given the reliance on SID in the caudal fold and carcass inspection at slaughter for infected farm detection in Fiji, QC should particularly focus on checking tuberculin potency and standardized training and competency testing of government meat inspectors.
4. Farmer cooperation with control and surveillance activities is vital for the success of animal disease programs. Active participation requires farmer knowledge of bTB risk and impact on cattle production and health, and farmer confidence that BTEC requirements are feasible and effective. Strengthening incentives, such as compensation for culling of positive animals will encourage more farmer cooperation. Effective communication about bTB via farmer targeted and general community campaigns is required to generate farmer action and community support. Clear messaging is proving challenging for bTB due to confusion about tuberculin skin test performance, the involvement of wildlife reservoirs in some countries, and local cultures and beliefs, particularly in countries where despite sustained control programs bTB remains endemic, such as Spain and the United Kingdom. Recent qualitative research involving farmers and veterinarians in Spain articulated the link between farmer non-participation in on-farm testing and distrust of official veterinary services and with farmer perception of little benefit to be gained from bTB freedom (28).

## Conclusion

The Government of Fiji has demonstrated sustained commitment to reduce bTB in the cattle population. The determination to succeed in a resource limited setting with challenging field conditions is to be commended. The history of bTB control elsewhere shows that the use of tuberculin tests (SID PPD-B and/or SICTT) needs to be relevant to the context (29) and the purpose of their application communicated clearly to avoid confusion and farmer disengagement (28). Guidance from the international animal health community is essential to inform refinement to the Fiji BTEC Strategy on the journey to a bTB-free Fiji.

## Author contributions

EB collated and analyzed the data for the retrospective study. RP assisted EB with collation and entry of the bTB data 1999–2014. LB, RP, and TT assisted with interpretation of the findings of the retrospective study. All authors contributed to the content and the preparation of this manuscript.

### Conflict of interest statement

The authors declare that the research was conducted in the absence of any commercial or financial relationships that could be construed as a potential conflict of interest. The reviewer MG declared a shared affiliation, with no collaboration, with one of the authors, EB, to the handling editor at time of review.
